# The role of cGAS-STING signaling in the liver and gastrointestinal system: from signaling networks to target intervention

**DOI:** 10.3389/fimmu.2025.1682992

**Published:** 2025-09-17

**Authors:** Zhengli Tan, Jiayue Shi, Shiyi Zhang, Yanyao Liu

**Affiliations:** ^1^ Department of Hepatobiliary Surgery, The First Affiliated Hospital of Chongqing Medical University, Chongqing, China; ^2^ College of Traditional Chinese Medicine, Chongqing Medical University, Chongqing, China

**Keywords:** cGAS-STING, liver diseases, gastrointestinal diseases, innate immune response, inflammation

## Abstract

The cyclic guanosine monophosphate-adenylate synthase (cGAS)-stimulator of interferon genes (STING) signaling pathway constitutes a fundamental mechanism through which the host innate immune system identifies cytoplasmic DNA. This pathway triggers a range of immune responses, notably the production of type I interferon (IFN-I), by detecting both exogenous pathogen-derived DNA and endogenous damage-associated DNA. It plays a pivotal role in anti-infective and anti-tumor responses, as well as in the maintenance of tissue homeostasis. Nevertheless, within the liver and gastrointestinal system—an environment persistently subjected to microbial and metabolic stress—the functionality of the cGAS-STING pathway demonstrates considerable complexity and context dependence. This review provides a comprehensive analysis of the molecular structure and activation mechanisms of the cGAS-STING signaling pathway. It systematically examines the variations in the pathway’s role across different disease contexts, cell types, and modes of stimulation. Furthermore, it synthesizes the latest therapeutic strategies targeting this pathway, offering a theoretical foundation and advanced insights for the development of more precise interventions for liver and gastrointestinal diseases.

## 1 Introduction

The innate immune system constitutes the host’s primary defense mechanism against both external pathogenic invasions and internal cellular damage. This system operates through a series of germline-encoded pattern recognition receptors (PRRs), which are responsible for identifying conserved pathogen-associated molecular patterns (PAMPs) and damage-associated molecular patterns (DAMPs) (1). Among the diverse array of PRRs, the cGAS-STING has been a pivotal component in the detection of cytoplasmic DNA, thereby becoming a central focus in contemporary immunological and disease-related research (2).

The liver and gastrointestinal tract collectively constitute a distinctive and functionally intricate organ system that functions not only as the central hub for digestion, absorption, and nutrient metabolism but also as the body’s primary defense against external pathogens and toxins. The intestinal mucosa is persistently exposed to substantial quantities of commensal microorganisms, pathogens, food antigens, and environmental toxins, necessitating its immune system to sustain a delicate equilibrium between immune tolerance and immune response (3). Receiving blood from the intestines through the portal vein circulation, the liver acts as a secondary defense against substances derived from the intestines. This unique anatomical and physiological configuration renders the liver and gastrointestinal system an exemplary model for investigating the cGAS-STING pathway. Firstly, this system represents a high-risk domain for various pathological processes, including infection, inflammation, metabolic disorders, and tissue damage, thereby offering diverse activation scenarios for the cGAS-STING pathway (4). Secondly, the stability of the liver and intestines is heavily dependent on the synergistic interaction between the immune and metabolic systems, with the cGAS-STING pathway identified as a critical link (5). Lastly, the system comprises a complex array of cell types, encompassing both parenchymal and non-parenchymal cells. The variations in expression and function of the cGAS-STING pathway among these cell types are pivotal in determining its ultimate physiological or pathological outcomes.

**Figure 1:**
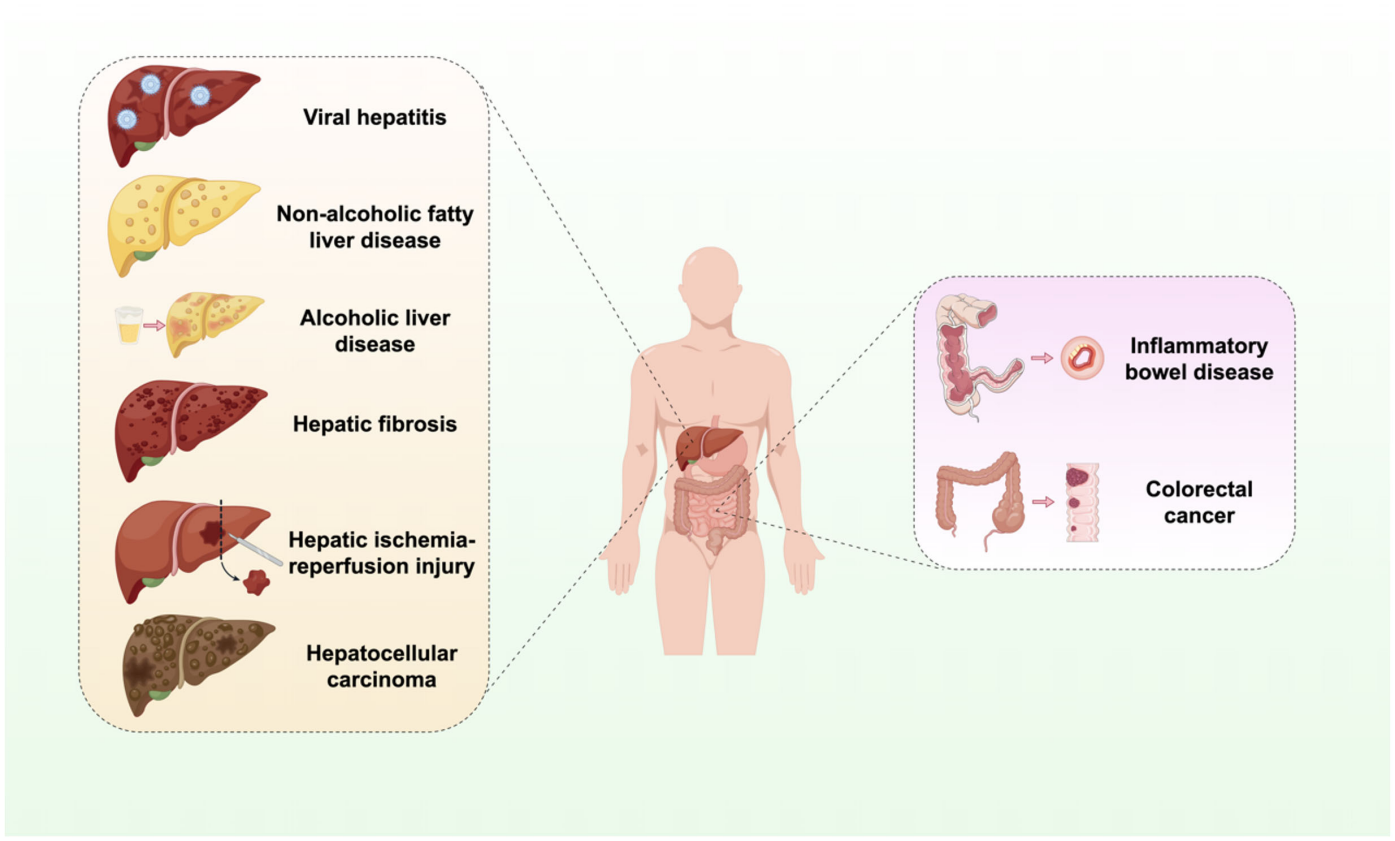
Activation of the cGAS-STING signaling pathway in liver and GI diseases. The cGAS-STING signaling pathway is integral to the processes of infection, inflammation, immunity, injury, and oncogenesis in the hepatic and GI systems. Infectious and inflammatory diseases include viral hepatitis, non-alcoholic liver disease, alcoholic liver disease. Autoimmune and injury-related diseases include liver fibrosis, IBD, and hepatic IRI. Neoplastic diseases include HCC and CRC.

This review seeks to systematically elucidate the role of the cGAS-STING signaling pathway in a range of hepatic and gastrointestinal diseases (**Figure 1**). It emphasizes the analysis of its distinct mechanisms within specific disease contexts and compiles recent advancements in targeted therapeutic strategies. The objective is to furnish a comprehensive reference for scientific inquiry and clinical translation in these related domains.

## 2 The structure and molecular mechanism of cGAS-STING signaling

A key component of the innate immune system’s defense against cytosolic DNA is the cGAS-STING signaling, functioning through precise molecular interactions that drive downstream inflammatory responses. Deciphering the structural basis and activation mechanisms of cGAS and STING is crucial for understanding their roles in disease pathogenesis and therapeutic targeting.

### 2.1 cGAS: structural features and immune activation paradigms

cGAS is a double-stranded DNA (dsDNA) sensor in the cytoplasm (6). It recognizes various pathogens that either contain or generate DNA throughout their life cycle (7). These pathogens include DNA viruses, retroviruses, bacteria, and parasites. cGAS functions are not limited to antimicrobial defense. Regardless of the sequence, cGAS is activated by dsDNA (8). Consequently, cGAS can be activated by its own DNA, including genomic DNA and mitochondrial DNA (mtDNA) (9). Genomic DNA or mtDNA can enter the cytoplasm due to cellular stress or environmental damage, activating cGAS and initiating an immune and inflammatory response. Endogenous cGAS predominantly resides within the nucleus, where it is closely associated with nuclear structures. This localization contributes to the maintenance of cGAS in a quiescent state, thereby preventing its interaction with self-DNA (10). In the absence of identifiable DNA, cGAS remains inactive and is believed to prevent unnecessary autoimmune responses (11). Upon binding to DNA, cGAS dimerizes and interacts with two dsDNA molecules to form a 2:2 complex, revealing two active enzymatic sites. The detection of foreign DNA by cGAS triggers structural alterations that increase the production of cGAMP (12). cGAMP is a dinucleotide that acts as an endogenous secondary messenger in the cGAS-STING pathway. It binds to the STING molecule on the endoplasmic reticulum (ER) membrane, inducing a conformational change that activates STING (11). Additionally, cGAMP activates STING in neighboring cells via gap junction proteins like Cx32 (13). Mouse liver cells can initiate antiviral responses in neighboring cells through interferon (IFN) secretion and direct crosstalk, including cGAMP transfer via gap junctions, to activate STING-dependent innate immunity (14).

### 2.2 STING as a central hub: molecular activation, trafficking, and signal transduction pathways

**Figure 2:**
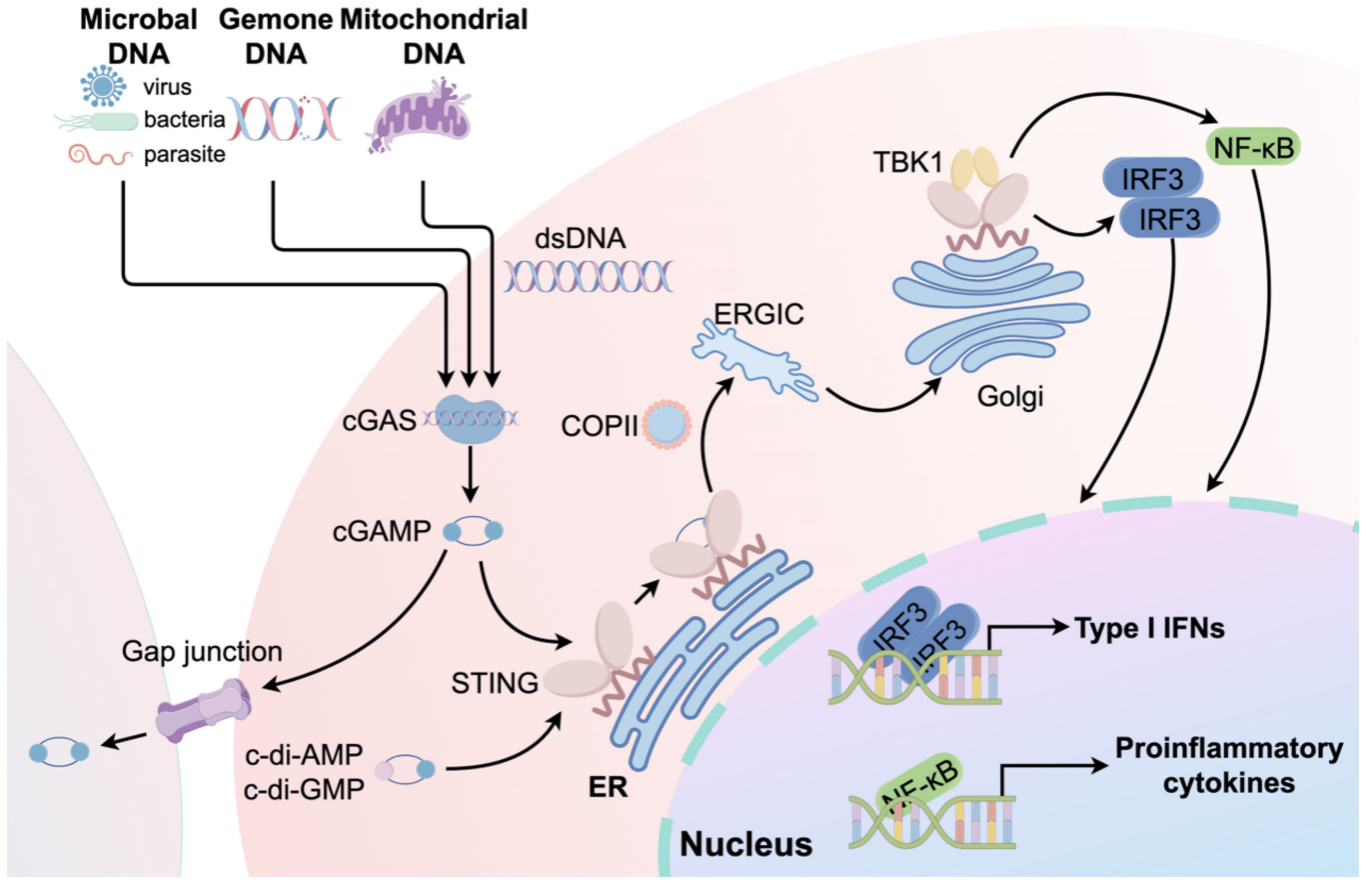
An overview of the cGAS-STING signaling pathway. The process begins when microbial DNA, genomic DNA, or mtDNA trigger cGAS, resulting in the creation of the second messenger, cGAMP. cGAMP subsequently stimulates STING, thereby activating the cGAS-STING-TANK-TBK1-IRF3/NF-κB signaling cascade. This activation results in the expression of type I IFNs and pro-inflammatory cytokines. In addition to cGAMP, cyclic di-adenosine monophosphate (c-di-AMP) or cyclic di-guanosine monophosphate (c-di-GMP) can directly activate STING. Furthermore, cGAMP can activate STING in neighboring cells through gap junctions.

STING is a transmembrane adaptor protein crucial for the natural immune signaling pathway. Five transmembrane regions are present, along with N-terminal and C-terminal domains. The N-terminal domain comprises four transmembrane helices (amino acids 1–152), whereas the C-terminal domain is primarily composed of a V-shaped ligand-binding domain oriented toward the cytoplasm, as well as a C-terminal tail (15). In addition to cGAS activating STING, numerous studies have confirmed that STING can directly sense cyclic di-AMP or cyclic di-GMP of bacterial origin in a cGAS-independent manner (16). STING transitions from the ER to the ER-Golgi intermediate compartment and then to the Golgi apparatus upon activation by these substances (17). ADP-ribosylation factor GTPases and Cytosolic coat protein complex II are crucial for STING dimerization and its transport to the ER-Golgi intermediate compartment and Golgi apparatus (18). STING recruits and phosphorylates TANK-binding kinase 1 (TBK1), which subsequently phosphorylates IFN regulatory factor (IRF3) and IκB kinase (IKK). As a result, IRF3 undergoes dimerization and moves to the nucleus, leading to the transcription and release of IFN-I and IFN-stimulated genes. Together with activated nuclear factor κB (NF-κB), these factors stimulate the release of chemokines and inflammatory cytokines, including interleukin-1β, interleukin-6 (IL-6), tumor necrosis factor-α (TNF-α), and type I IFNs (IFN-α and IFN-β) (**Figure 2**) (19).

## 3 cGAS-STING in infection and inflammation

As a central component of anti-infective immunity, the cGAS-STING pathway plays a pivotal role in mediating responses to pathogen invasion and associated inflammatory processes within the liver and gastrointestinal system. Nevertheless, its function is not uniform; instead, it demonstrates highly specific mechanisms and effects across various disease contexts.

### 3.1 Viral hepatitis

Viral hepatitis encompasses a group of infectious diseases instigated by diverse hepatitis viruses, predominantly marked by liver inflammation and necrosis. Notably, the hepatitis B virus (HBV) and hepatitis C virus (HCV) are principal agents responsible for chronic hepatitis, cirrhosis, and hepatocellular carcinoma (HCC), thereby constituting a significant global public health concern (20, 21). The interplay between the cGAS-STING pathway and hepatitis viruses exemplifies the enduring and complex “arms race” between host defenses and pathogenic strategies.

As a DNA virus, the HBV genome would typically serve as a direct substrate for cGAS recognition, thereby initiating a robust antiviral immune response. Nevertheless, the capacity of HBV to establish chronic infection is primarily attributed to its evolution of a sophisticated and efficient immune evasion strategy, which suppresses the activation of the cGAS-STING pathway through a variety of mechanisms (22). Firstly, HBV directly disrupts critical nodes within this pathway via its viral proteins. Specifically, the HBV polymerase (Pol) not only fulfills its conventional role as a reverse transcriptase but also functions as an immunosuppressive agent. HBV Pol is capable of physically binding to the STING protein, with its reverse transcriptase and RNase H domains being essential for facilitating this inhibitory interaction. This binding does not result in the degradation of the STING protein; rather, it specifically obstructs the K63-linked polyubiquitination modification, which is vital for STING activation (23). Secondly, HBV compromises the host’s innate DNA sensing capabilities at their origin. Recent studies have demonstrated that HBV utilizes its encoded microRNA, designated as HBV-miR-3, to precisely modulate the expression of cGAS. Through post-transcriptional regulatory mechanisms, HBV-miR-3 specifically binds to the 3’ untranslated region of cGAS mRNA, inhibiting the translation of the cGAS protein. This interaction fundamentally impairs the cell’s capacity to detect viral DNA (22). Moreover, other HBV proteins, such as the HBx protein, have been implicated in the inhibition of this pathway through various mechanisms, including the mediation of cGAS ubiquitination and degradation or the disruption of downstream signaling molecules (24). TRIM29, a constituent of the TRIM protein family, has been demonstrated to ubiquitinate and subsequently degrade STING, thus attenuating the production of IFN-I (25). Recent studies have elucidated its significant involvement in viral myocarditis and enterovirus infections, both of which are viral infectious diseases (26, 27). Consequently, the potential role of TRIM family protein members in the context of HBV warrants further investigation. Additionally, as an “invisible virus,” HBV encapsulates its viral genome within core particles upon cellular entry, potentially physically obstructing cGAS recognition and constituting another passive evasion strategy (28, 29).

In contrast to HBV, HCV is an RNA virus, and its genome is not typically detected by the DNA sensor cGAS (30). Nevertheless, accumulating evidence indicates that the cGAS-STING signaling pathway is inadvertently activated during HCV infection, contributing to the antiviral response and suggesting a non-classical mechanism of immune recognition (31). This activation is not a result of direct detection of viral RNA but is instead triggered by cellular stress associated with HCV infection. The replication and protein expression of HCV impose considerable metabolic and organellar stress on host cells, particularly affecting the mitochondria. HCV infection can result in mitochondrial dysfunction, morphological alterations, and increased oxidative stress, which ultimately cause mtDNA to escape from the mitochondrial matrix into the cytoplasm (32). These mtDNA fragments serve as typical DAMPs for cGAS, which can effectively detect them and activate the STING pathway (33). Experimental evidence demonstrates that in the initial phases of HCV infection, there is an upregulation of intracellular cGAS expression, an elevation in cGAMP levels, and observed colocalization of cGAS and STING with mitochondria. Gene knockout or RNA interference techniques that suppress cGAS or STING expression lead to a significant increase in HCV replication and a reduction in IFN-β production. These findings underscore the critical role of this signaling pathway in the regulation of HCV infection (34).

Due to the protective role of STING pathway activation in mitigating HBV-induced liver damage, researchers are actively screening and developing STING agonists or small molecules with STING agonist properties to combat HBV. Recent research indicates that Schisandra C promotes the cGAS-STING signaling pathway, thereby exhibiting antiviral effects in HBV-infected mice (35). RVX-208 stimulates apolipoprotein A-I and reduces HBV particle production through the activation of the cGAS-STING pathway (36). The topoisomerase II (TOPII) inhibitor, daunorubicin, inhibits HBV production by activating the endogenous cGAS pathway in NKNT-3/NTCP cells (37). Further research is required to develop drugs for the treatment of HBV infection.

The distinct interaction patterns between HBV and HCV with the cGAS-STING pathway underscore the intricate nature of virus-host coevolution. HBV, as a DNA virus, encounters substantial selective pressure to be directly identified by cGAS, resulting in the development of sophisticated, multi-layered inhibitory mechanisms. Conversely, HCV, an RNA virus, indirectly activates this otherwise unrelated immune pathway by disrupting cellular metabolism. This illustrates the adaptability of the host immune system in leveraging cellular damage signals to identify a wide range of threats.

### 3.2 NAFLD

Non-alcoholic fatty liver disease (NAFLD) represents a chronic hepatic condition intricately linked with metabolic syndrome (38). Its incidence has escalated in tandem with the global rise in obesity and type 2 diabetes, establishing it as the predominant chronic liver disease worldwide. Within the pathophysiological framework of NAFLD, the cGAS-STING pathway transitions from its traditional role as an antagonist to infection to become a pivotal mediator of sterile inflammation. The underlying mechanisms are primarily centered on metabolic stress, mitochondrial impairment, and the heterogeneous responses of various hepatic cellular subpopulations.

The fundamental pathophysiological basis of NAFLD is the excessive intracellular accumulation of free fatty acids within hepatocytes, a phenomenon termed lipotoxicity. This lipotoxicity imposes considerable stress on organelles, particularly mitochondria, which are integral to energy metabolism. Under conditions of sustained metabolic burden, hepatic mitochondrial dysfunction ensues, leading to the release of mtDNA into the cytoplasm. This cytoplasmic mtDNA functions as an endogenous DAMP, serving as a potent activator of the cGAS-STING pathway (4). In patients with NAFLD and in animal models induced by high-fat diets, significant elevations in mtDNA levels in both liver and blood have been observed, with these elevations showing a positive correlation with disease severity (39). Similarly, the accumulation of microbial DNA in NAFLD contributes to the progression of hepatic steatosis and fibrosis. Furthermore, iron overload can aggravate chronic liver disease by triggering inflammation through the cGAS-STING signaling pathway (40, 41).

A notable characteristic of cGAS-STING pathway activation in NAFLD is its pronounced cell type specificity. Extensive research has demonstrated that in the normal livers of adult mice and humans, hepatocytes exhibit minimal or undetectable levels of STING protein expression (42). This suggests that even if hepatocytes generate substantial amounts of cytoplasmic mtDNA, they are unable to effectively initiate downstream responses via the STING pathway, thus remaining in a “signal-silent” state. In contrast, non-parenchymal cells in the liver, particularly Kupffer cells and other myeloid-derived macrophages, display high levels of STING protein expression. When hepatocytes are damaged, they release mtDNA as a “distress signal,” which is subsequently “received” by neighboring Kupffer cells, leading to the activation of their STING pathway. Upon activation, Kupffer cells predominantly secrete pro-inflammatory cytokines such as TNF-α and IL-6 through the NF-κB signaling pathway, rather than the IRF3-IFN-I pathway (43). These cytokines subsequently exert effects on hepatocytes, leading to increased lipid accumulation, insulin resistance, and apoptosis. Concurrently, they activate hepatic stellate cells (HSCs), thereby facilitating the advancement of fibrosis. This process establishes a self-perpetuating cycle that perpetuates the progression from NAFLD to non-alcoholic steatohepatitis (NASH) and liver fibrosis.

Recognizing the significant impact of the cGAS-STING pathway on inflammation and fibrosis in NAFLD/NASH, targeting this pathway to inhibit its activity has emerged as a hopeful therapeutic strategy. RNF13 effectively inhibits lipid accumulation, inflammation, and metabolic dysfunction during NASH progression. It supports TRIM29 stabilization via K63-linked ubiquitination, which permits TRIM29 to ubiquitin-dependently break down STING and prevent the irregular activation of downstream signaling pathways (44). Licorice extract can ameliorate liver inflammation and fibrosis in NASH mice by disrupting STING oligomerization and inhibiting the cGAS-STING pathway (45). In summary, the pharmacological inhibition of STING activation could be used as a therapeutic approach against NAFLD/NASH, facilitating the development of new treatments targeting this pathway.

### 3.3 ALD

Alcoholic liver disease (ALD) is marked by liver injury due to long-term excessive drinking and is a leading cause of cirrhosis and alcohol-related deaths worldwide. The pathogenesis of ALD is primarily driven by the direct toxicity of alcohol and its metabolites, oxidative stress, nutritional imbalances, and dysbiosis of the gut microbiota (46). Within the context of ALD, the cGAS-STING pathway plays a crucial pro-inflammatory role, with its activation mechanism being more intricate than that observed in NAFLD.

**Figure 3:**
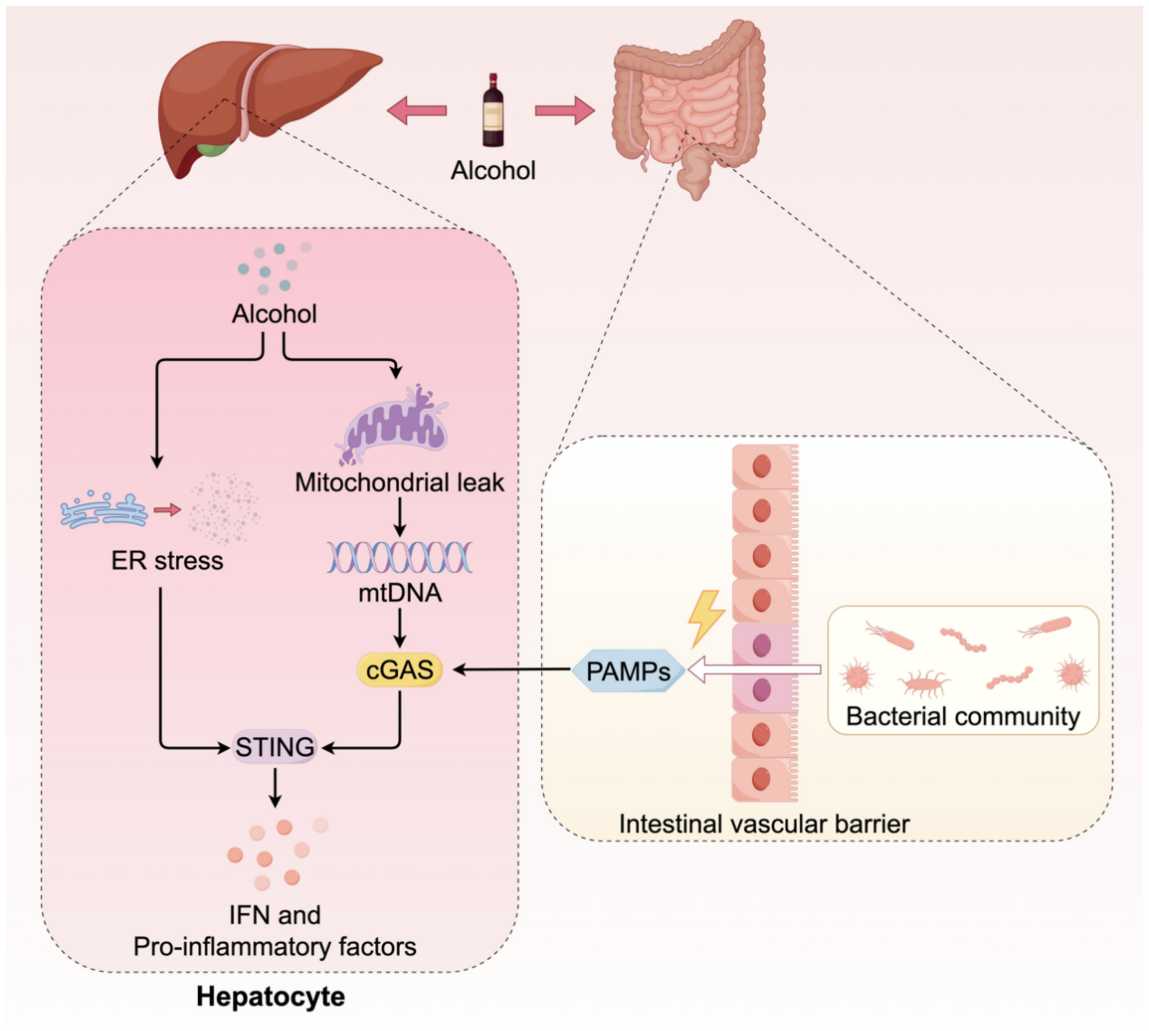
The cGAS-STING signaling in ALD. The activation of the cGAS-STING pathway in ALD is attributed to multiple sources. Firstly, metabolites of alcohol induce mitochondrial damage, resulting in the release of mtDNA. Furthermore, alcohol compromises the integrity of the gastrointestinal tract barrier, facilitating the translocation of gut microorganisms into the liver. The dsDNA from these microorganisms serves as a coactivator for cGAS. Additionally, the metabolic processing of alcohol can induce ER stress, which directly activates the downstream signaling of STING.

The activation of the cGAS-STING pathway in ALD is attributed to the synergistic interaction of at least three contributing factors (**Figure 3**). Analogous to NAFLD, the hepatic metabolism of alcohol in ALD results in the production of substantial amounts of ROS, which induces severe oxidative stress and mitochondrial damage. This process facilitates the release of mtDNA into the cytoplasm, thereby activating cGAS. In both ALD patients and alcohol-fed animal models, significantly elevated levels of mtDNA-rich particles have been detected in serum, correlating with the activation of the cGAS-STING pathway (47, 48). Alcohol exerts a direct deleterious impact on the gastrointestinal mucosa, resulting in compromised intestinal barrier function. This compromise facilitates the translocation of bacteria and their components from the intestine through the intestinal wall, subsequently entering the liver via the portal vein. These exogenous bacterial DNAs serve as potent PAMPs, which are recognized by cGAS in hepatic immune cells, thereby intensifying the activation of the STING pathway (49). The metabolic processing of alcohol significantly disrupts the protein folding capabilities of the ER, inducing severe ER stress. Studies have demonstrated that ER stress can independently function as a non-canonical signal to activate STING, leading to the phosphorylation of IRF3 and subsequently inducing mitochondrial apoptosis through interaction with the pro-apoptotic protein Bax, ultimately resulting in hepatocyte death (50). Beyond multiple activation triggers, the STING signaling pathway in ALD also displays a distinctive amplification mechanism. Recent research has demonstrated that cGAMP can be transmitted to adjacent, undamaged hepatocytes via intercellular gap junctions. This transmission amplifies localized damage into broader inflammatory and apoptotic responses, thereby significantly accelerating the progression of ALD (47). Within the context of ALD, the cGAS-STING pathway functions as a pivotal signal integration and amplification platform. It converges insults from metabolic damage, gut microbiota, and organelle stress, thereby exacerbating the deleterious effects through intercellular communication. This collective action drives the severe inflammatory response and hepatocyte apoptosis characteristic of ALD.

Although no STING inhibitors specifically targeting ALD have yet progressed to clinical trials, potential therapeutic targets, such as direct inhibition of STING or cGAS, blockade of the endoplasmic reticulum stress-STING axis, inhibition of intercellular gap junctions, and enhancement of intestinal barrier function, present promising avenues for the development of effective ALD treatments.

## 4 cGAS-STING in autoimmunity and injury

### 4.1 Hepatic fibrosis

Chronic liver disease is often characterized by liver fibrosis, a compensatory response secondary to tissue repair following various chronic liver injuries (51). A lack of effective interventions can lead to cirrhosis, liver failure, or HCC. Liver fibrosis is primarily caused by the excessive accumulation of extracellular matrix (EM). Activated HSCs are the primary contributors to EM production in fibrotic livers (52).

Engaging the cGAS-STING pathway might elevate liver inflammation, promote fibrosis, harm liver sinusoidal endothelial cells (LSECs), and hasten microthrombi development in hepatic sinusoids (53). Recent studies demonstrate that fibrosis is not solely driven by inflammation, with hepatocyte death emerging as a key factor during liver fibrosis (54). IRF3 contributes to liver fibrosis by promoting hepatocyte apoptosis. Accordingly, liver fibrosis may be mediated by inflammation and hepatocyte death.

Eliminating activated HSCs to prevent excessive EM deposition is an effective strategy for treating liver fibrosis. Atractylenolide III (ATR III) mitigates liver fibrosis by suppressing the cGAS/NF-κB pathway in HSCs, which subsequently inhibits the senescent cell-derived senescence-associated secretory phenotype (SASP) and decreases inflammation (55). Furthermore, managing HSC proliferation and triggering HSC apoptosis alone is insufficient to eliminate activated HSCs. HSC senescence contributes to liver fibrosis pathogenesis. The induction of HSC senescence could be a potential strategy for the removal of activated HSCs from the liver (56). HSC senescence limits proliferation, reduces EM secretion, and activates SASPs to attract NK cells for clearance, thereby preventing over-proliferation and transdifferentiation into fibroblasts (57). Moreover, some scholars have investigated related treatments. This approach integrates immune clearance with its improvement through targeted manganese (Mn) delivery, a cGAS-STING stimulator facilitated by albumin-mediated transcytosis. Upon endocytosis in activated HSCs, Mn@ALB NPs by the self-assembly of Mn^2+^ and human albumin, are lysosomally degraded, releasing Mn^2+^ that activates the cGAS-STING pathway, leading to SASP factor production. SASP factors and Mn^2+^ promote NK cell activation and cytotoxicity. The improved immune clearance of senescent HSCs can reverse liver fibrosis (58). Through the cGAS-STING pathway, Oroxylin A from Scutellaria baicalensis affects ferroptosis-induced HSC aging, helping to ease liver fibrosis (59). Oroxylin A not only regulates iron autophagy but also induces HSC aging by inhibiting cGAS gene methylation by suppressing methionine metabolite production (60). The reason why the cGAS-STING pathway plays an inhibitory role in liver fibrosis in the abovementioned studies may be attributed to its role in HSCs and not in other cells.

### 4.2 Inflammatory bowel disease

IBD, which is also referred to as CD or UC, is a long-term condition marked by inflammation in the gastrointestinal tract (61). IBD pathophysiology is typically viewed as an excessive and dysregulated immune response characterized by inflammation (62).

**Figure 4:**
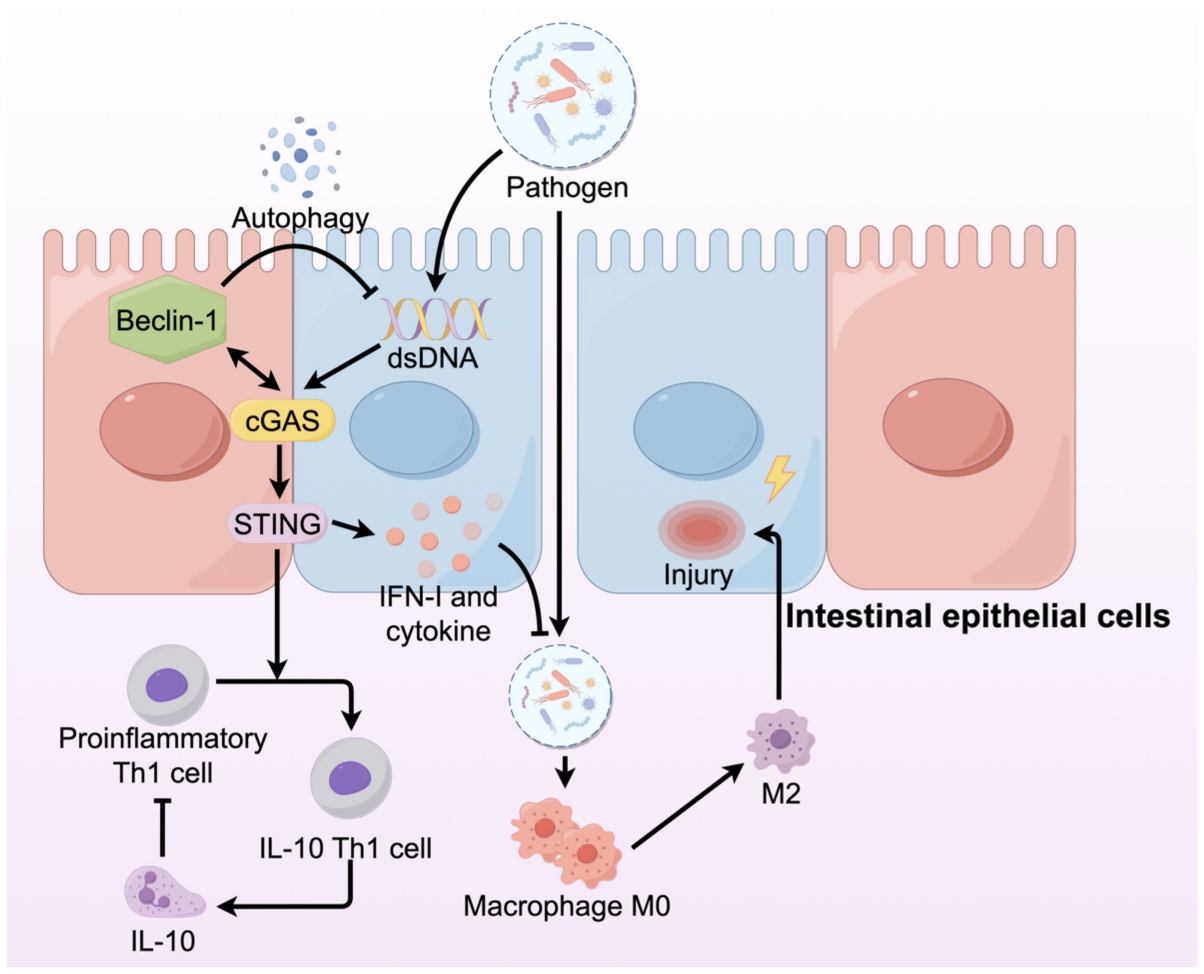
The cGAS-STING signaling pathway in IBD plays different roles in different cells. In IBD, the integrity of intestinal architecture is compromised, resulting in increased permeability. This disruption allows pathogens, including bacteria, to infiltrate the intestinal lamina propria, where they activate macrophages and promote their differentiation into the M2 phenotype, subsequently damaging the intestinal mucosa. Within intestinal epithelial cells, the cGAS-STING signaling pathway is activated, producing IFNs and cytokines that prevent pathogen invasion.

STING has recently emerged as a key regulator of intestinal homeostasis by activating pro-inflammatory IFN-I and cytokines that must be tightly controlled to maintain intestinal balance (63). STING activation requires a balance of regulatory mechanisms to prevent excessive inflammation. Both humans suffering from IBD and mice with colitis show increased STING expression in their immune and epithelial cell lineages (64). However, studies of the effect of this upregulation on IBD have revealed that STING may play different roles in different cell types (**Figure 4**). Moreover, mice with STING deficiency, specifically in macrophages, neutrophils, or DCs, exhibited reduced colonic inflammation and polyp formation when treated with AOM/DSS compared to STING knockout mice (65). The varying outcomes might stem from the dual functions of STING: it acts protectively in intestinal epithelial cells while promoting inflammation in myeloid cells. In patients with IBD, dysbiosis leads to the accumulation of STING in intestinal myeloid cells, thereby inducing intestinal inflammation (64). However, STING plays an opposite role in intestinal epithelial cells. Within the intestinal environment, immune, epithelial, and endothelial cells express the aromatic hydrocarbon receptor (AHR), which is crucial for preserving balance between the host and the gut microbiota (66, 67). Activation of AHR within the intestinal environment facilitates metabolic processes, reduces inflammation, and contributes to the restoration of the intestinal barrier in IBD, all of which are dependent on the presence of STING1. Consequently, the lack of STING1 compromises the AHR-mediated anti-inflammatory effects through various mechanisms, indicating that nuclear STING likely plays a key role in sustaining intestinal immune tolerance in a stable colon environment, while the cGAS-STING-IRF3 pathway is mainly responsible for inducing hyperinflammation in the presence of damage-associated molecular patterns(DAMPs) (68, 69). Downstream of STING signaling, its regulatory influence is manifested by the modulation of inflammatory cells and mediators. The pathogenic activity of Th1 cells is lessened by STING, which changes them into Th1 cells that secrete IL-10. In the absence of STING, T cells contribute to the exacerbation of colitis, whereas Th1 cells pretreated with STING agonists ameliorate the severity of colitis via IL-10 mediation (70). In addition to its role in inflammation, autophagy contributes to the disruption of normal intestinal epithelial homeostasis. The interaction between the NTase domain of cGAS and Beclin-1 in the presence of dsDNA leads to the suppression of 2’3’-cGAMP and aids in the autophagy-driven degradation of pathogenic DNA (17).

Inhibiting the overactive cGAS-STING, which is crucial for intestinal homeostasis and inflammation regulation, could be an effective therapeutic strategy for IBD. RU.521, the first selective cGAS inhibitor, was identified using high-throughput screening and subsequent structural enhancement. Immunoblot analysis demonstrated that intraperitoneal administration of RU.521 in a mouse model effectively targeted the cGAS-STING signaling pathway, thereby ameliorating colitis symptoms (71). Recent research indicates that programmable nanomicelles, engineered for colitis treatment and incorporating RU.521, have demonstrated promising results. The STING-inhibiting micelles (SIM) were designed to target inflammatory sites and release drugs upon encountering ROS. Oral SIM administration in mice alleviated colitis by downregulating STING expression, suppressing pro-inflammatory cytokine secretion, promoting weight recovery, preventing colon shortening, and restoring colonic epithelium (72). Researchers have developed a framework combining computational screening, microbiome interference, gene knockdown technology, and both *in vitro* and *in vivo* validation to predict biomarkers, identify host-microbe interaction targets, and repurpose drugs for IBD. The team successfully confirmed the efficacy of brefeldin A, a membrane protein transporter inhibitor (73). This approach is anticipated to serve as an efficient drug screening method in the future.

### 4.3 Hepatic ischemia-reperfusion injury

Hepatic ischemia-reperfusion injury (HIRI) frequently occurs in clinical contexts such as liver transplantation, hepatic resection, and traumatic injury, and it constitutes a significant contributor to postoperative liver dysfunction and failure (74). Within this sterile injury model of HIRI, the cGAS-STING signaling pathway reveals its most intricate and unexpected characteristics, with the primary components, cGAS and STING, demonstrating distinct and opposing roles, thereby creating a unique pathophysiological paradox.

During HIRI, hepatocytes experience significant damage due to hypoxia and oxidative stress, leading to the release of substantial amounts of mtDNA. It can be recognized by Kupffer cells, which subsequently activate the cGAS-STING pathway. This process recruits inflammatory cells, such as neutrophils, to infiltrate the tissue, thereby exacerbating inflammatory damage and hepatocyte death. In HIRI models, mice deficient in STING demonstrate a significant reduction in liver damage and improved hepatic function (75). Conversely, cGAS knockout mice exhibit markedly worsened liver damage following HIRI, indicating that cGAS exerts a protective role in HIRI that is independent of its downstream effector molecule, STING. Research has demonstrated that under HIRI-induced stress conditions, cGAS activation in hepatocytes facilitates autophagosome formation, thereby maintaining intracellular homeostasis (76).

The dual role of the cGAS-STING pathway in HIRI necessitates more sophisticated treatment strategies. Simple cGAS inhibitors may inadvertently impede its beneficial autophagic function, potentially leading to adverse effects. Consequently, more nuanced intervention strategies are required. Direct inhibition of STING or its downstream signaling pathways represents a clearly effective approach. For instance, compounds such as dimethyl fumarate have demonstrated efficacy in mitigating HIRI by disrupting the interaction between STING and its downstream effectors TBK1/IRF3 (77). Another viable strategy involves inhibiting the release of mtDNA. Agents targeting the mitochondrial permeability transition pore or voltage-dependent anion channel, such as IMT1B, can curtail mtDNA leakage into the cytoplasm, thereby preventing the activation of STING at its source (75). The optimal therapeutic approach may involve the development of drugs capable of selectively blocking signal transduction between cGAS and STING, while preserving the STING-independent pro-autophagic function of cGAS. This would enable precise therapeutic interventions that effectively “discard the dregs and retain the essence.”

## 5 cGAS-STING in cancer

### 5.1 Hepatocellular carcinoma

Hepatocellular carcinoma (HCC) is the most common type of primary liver cancer worldwide and is known for its high mortality rate. Its development is strongly linked to sustained inflammation and fibrosis associated with chronic liver disease (78). Within HCC, the cGAS-STING signaling pathway demonstrates a notable duality in function, contingent upon the temporal dynamics of signal activation and the specific cellular context.

During the chronic liver disease stage that precedes HCC, the persistent, low-level activation of the cGAS-STING pathway exhibits tumorigenic properties. As previously discussed, chronic inflammation driven by DAMPs and PAMPs, such as mtDNA or bacterial DNA, continuously stimulates hepatic immune cells and HSCs. This stimulation creates a microenvironment conducive to fibrosis, angiogenesis, and cell proliferation, thereby establishing the “soil” necessary for the malignant transformation of hepatocytes (4, 49). Furthermore, prolonged STING signaling can lead to immune cell exhaustion and facilitate early tumor cell evasion of immune surveillance by upregulating immune checkpoint molecules, such as PD-L1 (79). Research has demonstrated that Tetrandrine can augment the efficacy of PD-1 immunotherapy in cancer by modulating the STING pathway. The incorporation of Chinese herbal medicine may serve as a significant strategy to prevent immune exhaustion (80). In contrast to chronic activation, acute and potent activation in established HCC serves as an effective antitumor strategy. The core mechanism of this strategy involves transforming an immune-suppressive “cold tumor” into an immune cell-infiltrated “hot tumor” (42). Upon the death or damage of tumor cells, which may result from therapeutic interventions like radiotherapy or chemotherapy, or due to intrinsic genomic instability, their DNA—including nuclear DNA fragments, micronuclei, and mtDNA—is released into the cytoplasm. This tumor-derived DNA is subsequently recognized by the cGAS within APCs, such as DCs, located in the tumor microenvironment, thereby activating the STING pathway, leading to the production of substantial quantities of IFN-I in DCs. As a pivotal immune regulatory factor, IFN-I facilitates the maturation, activation, and antigen cross-presentation capacity of DCs, enhances the expression of MHC-I molecules on the surface of tumor cells—thereby rendering them more susceptible to recognition by T cells—and directly augments the proliferation and cytotoxic functions of CTLs (81, 82).

Due to their robust capacity to activate anti-tumor immune responses, STING agonists have emerged as a highly promising avenue of research within the domain of tumor immunotherapy, particularly in the treatment of HCC. A variety of synthetic cyclic dinucleotide (CDN) analogues, such as ADU-S100, MK-1454, and BMS-986301, along with non-CDN small-molecule agonists, have been developed and have shown efficacy in preclinical models of HCC, either as standalone treatments or in combination with other therapies (83). The prevailing trend in this research area is combination therapy, wherein STING agonists are utilized alongside conventional treatments like immune checkpoint inhibitors (ICIs), radiotherapy, and radiofrequency ablation to achieve synergistic effects and to overcome drug resistance. However, the direct systemic administration of STING agonists is associated with the risk of inducing severe cytokine storms and systemic toxic side effects (42). Additionally, their hydrophilic nature and instability pose significant challenges to their delivery efficiency within the body (84). To address these challenges, nanodelivery systems have been developed. By employing carriers such as lipid nanoparticles, polymer micelles, and exosomes, STING agonists can be encapsulated and directed to specific tissues, including the liver and tumor sites. This approach not only substantially elevates drug concentration within the tumor microenvironment but also enhances its uptake by APCs, thereby optimizing antitumor efficacy while reducing systemic toxicity (85–87).

### 5.2 Colorectal cancer

CRC represents about 10% of global cancer diagnoses and deaths, posing a major health threat worldwide (88). CRC patients with elevated STING expression exhibit enhanced intra-tumoral infiltration of CD8+ T cells and reduced lymphatic vessel infiltration during the early stages of the disease. These individuals experience prolonged overall survival and recurrence-free survival in comparison to patients with low STING expression (89).

Current research extensively explores the cGAS-STING signaling pathway in CRC, and the potential mechanisms are elucidated in this study by examining each module of the pathway. In tumor cells, the main cGAS activators are DNA after tumor cell damage and mtDNA. Research has focused on improving tumor cell damage to trigger the cGAS-STING signaling pathway and activate the innate immune response. In cancer cells, RT-induced endogenous production of IFN-I primarily relies on the cGAS/STING pathway activated by cytoplasmic dsDNA, boosting cancer immunogenicity and strengthening antitumor immune responses, leading to better treatment outcomes (90). The TOPI inhibitor SN38 activates the cGAS-STING pathway and triggers an immune response via DNA damage. Combining lovastatin, which impedes DNA damage repair, elevates DNA damage and enhances cGAS-STING pathway activation, thereby amplifying immunotherapy efficacy (91). Ataxia telangiectasia mutated kinase and ataxia telangiectasia and Rad3-related (ATR) kinase are crucial for the cellular response to DNA damage. The combination of irradiation and ATR inhibitors (ATRi) improves the classical cGAS-STING-pTBK1/pIRF3 pathway by elevating cytoplasmic dsDNA levels (92). Poly (ADP-ribose) polymerases PARP-1 and PARP-2 function as DNA repair mechanisms in cancer cells, and treatment with PARP inhibitors (PARPi) improves the cytoplasmic accumulation of IR-induced dsDNA (93). Limiting the nutrient supply to tumor cells may cause mtDNA accumulation in the cytoplasm and induce direct mitochondrial damage. Restriction of serine availability in CRC cells induces mitochondrial dysfunction, resulting in the accumulation of mtDNA in the cytoplasm. This accumulation subsequently activates the cGAS-STING signaling pathway, culminating in the secretion of IFN-I (94). Methionine deprivation modifies the nutrient metabolic pathway by inhibiting methylation, thereby promoting cGAS activity and tumor immunity (95).

**Figure 5:**
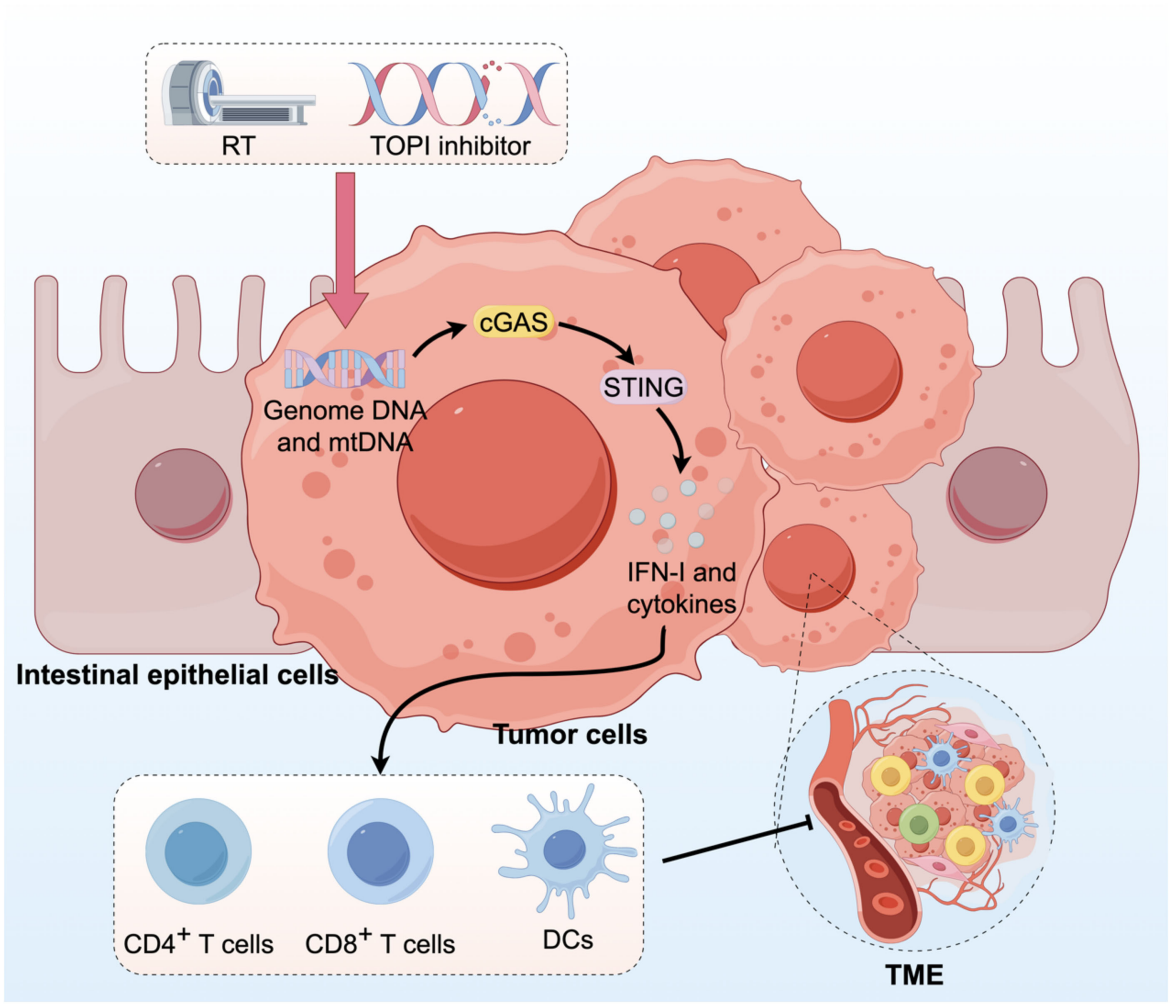
Activation of the cGAS-STING pathway in CRC can augment innate immune responses. RT and administration of TOPI inhibitors induce tumor damage, resulting in the release of dsDNA, which subsequently activates the cGAS-STING pathway. This activation enhances the infiltration of CD4+ T cells, CD8+ T cells, and DCs into the TME.

The downstream cGAS-STING pathway is linked to immune cells, including IFN, which plays a crucial role. The combination of irinotecan dioxide nanorods and RT activates the cGAS/STING pathway in CRC, resulting in the enhanced infiltration of CD4+ T cells, CD8+ T cells, and DCs within the TME (**Figure 5**) (96). Some studies demonstrate that persistent activation of the cGAS-STING pathway in CRC may lead to downstream signal rewiring in cancer cells, thereby fostering a pro-metastatic TME (97). This suggests that the role of cGAS-STING in CRC requires further research. In conclusion, combining STING agonists or bacteria that boost cGAS-STING signaling with conventional antitumor therapies could be a potential strategy for CRC treatment.

## 6 Deeper insights into the significance and function of cGAS-STING

The cGAS-STING signaling pathway demonstrates markedly diverse and sometimes contradictory functions across various hepatic and gastrointestinal diseases. Its roles span from serving as an antiviral “guardian” to acting as a pro-inflammatory “catalyst,” and from functioning as an anti-tumor “sword” to facilitating a pro-carcinogenic “breeding ground.” A comprehensive investigation into the underlying causes of these functional disparities, along with a strategic framework for the precise application of this pathway, is essential for advancing both fundamental research and clinical translation.

The cGAS-STING pathway, often perceived as a straightforward “DNA sensing-IFN/inflammation production” linear model, is in fact modulated by a multitude of factors within complex biological systems, resulting in diverse outcomes. The variability in its functional roles can be attributed to several levels of regulation. Foremost, the cell type in which the pathway is activated plays a pivotal role in determining its function. Activation within immune cells generally triggers classical immune responses. For instance, in the context of NAFLD, Kupffer cells are the primary responders to STING activation. This activation induces the release of pro-inflammatory mediators via the NF-κB pathway, thereby promoting sterile inflammation (43). Conversely, in tumor immunotherapy, the activation of the STING pathway in DCs is crucial for the initiation of adaptive anti-tumor immunity (81, 82). The activation of cGAS in somatic cells frequently facilitates cell-specific non-canonical functions. In the context of ALD, ER stress in hepatocytes directly activates the STING, leading to apoptosis rather than a pronounced IFN response (50). In liver fibrosis, the activation of STING in HSCs is fundamentally necessary for their transdifferentiation into myofibroblasts (55). In HIRI, cGAS within hepatocytes plays a STING-independent role in promoting protective autophagy (76). Furthermore, the characteristics of the DNA “messengers” that activate the cGAS-STING pathway significantly affect downstream outcomes. PAMPs, such as viral DNA, typically elicit a rapid and intense IFN-I response aimed at swiftly eradicating infections (22). In contrast, DAMPs, such as mtDNA, often provoke sustained, low-intensity responses that are more likely to result in chronic, NF-κB-driven pathological inflammation, as observed in NAFLD and ALD (4, 47).In conclusion, the temporal dynamics of signal activation serve as a pivotal factor distinguishing between “beneficial” and “detrimental” pathways, particularly in the context of tumorigenesis. Acute activation is generally advantageous; for instance, in antiviral or antitumor immunity, transient and robust activation of the STING pathway through pharmacological agents, such as STING agonists, or radiotherapy can effectively stimulate the immune system to eradicate pathogens or tumor cells (42). Conversely, chronic activation is frequently deleterious. In conditions such as chronic liver disease or IBD, sustained damage signals, including mitochondrial DNA or gut microbiota DNA, result in prolonged low-level activation of the cGAS-STING pathway. This persistent stimulation not only perpetuates tissue inflammation and fibrosis, thereby creating a conducive environment for tumorigenesis, but also may induce PD-L1 expression and immune cell exhaustion. This ultimately leads to immune tolerance and escape, thereby facilitating tumor progression (79).

The intricate and dual nature of the cGAS-STING pathway poses significant challenges for therapeutic interventions, while simultaneously indicating potential avenues for future development. Furthermore, addressing the toxicity associated with STING agonists presents a significant challenge. The activation of the STING pathway results in the overproduction of proinflammatory cytokines, potentially leading to severe adverse effects, including fever and chills. The risk is exacerbated with systemic administration, underscoring the necessity to optimize dosing strategies and develop more selective STING activators to reduce off-target effects (84). Emerging therapies must transcend simplistic approaches of “systemic activation” or “systemic inhibition.” It is essential to advance technologies capable of precisely delivering STING agonists or inhibitors to targeted cell types (85). In the context of employing STING agonists for cancer therapy, it is crucial to ascertain the optimal timing, dosage, and frequency of administration to establish an effective therapeutic window, thereby mitigating the risk of immune exhaustion or toxic side effects due to overactivation. This endeavor may necessitate the utilization of biomarkers to dynamically monitor the immune status of patients (42). Future drug development should transcend the binary approach of simple “on” or “off” switches. It is imperative to explore more sophisticated regulatory strategies, such as the design of allosteric modulators capable of selectively inhibiting the NF-κB pathway downstream of STING while sparing the IFN-I pathway, or vice versa. Moreover, in-depth investigation into the STING-independent roles of cGAS, such as its involvement in autophagy regulation during HIRI, alongside the discovery of molecules that can specifically modulate these functions, has the potential to unveil novel avenues for therapeutic intervention (76).

## 7 Conclusion

The cGAS-STING signaling pathway, as a fundamental component of the innate immune system, plays a significant and intricate role in the physiological and pathological processes of the liver and gastrointestinal system. It functions not only as a robust defense mechanism against viral infections but also as a sensitive detector of cellular stress and tissue damage. This review systematically elucidates that the functions and effects of this pathway are dynamic and are meticulously regulated by the disease context, cell type, stimulus signals, and timing of activation. In the context of antiviral immunity, it acts as both a potent mechanism for the host to eliminate viruses and a target that viruses attempt to suppress to establish persistent infection. In metabolic diseases such as NAFLD and ALD, it serves as the central driver of sterile inflammation induced by metabolic disorders. During processes of tissue damage and repair, it can facilitate fibrosis and inflammatory damage through STING-dependent mechanisms, while also exerting protective effects via cGAS-dependent non-canonical pathways. During tumor development, the STING pathway exhibits dual roles: it acts as a protective agent against cancer during acute activation but may promote tumorigenesis under chronic stimulation.

A comprehensive understanding of this context-dependent behavior presents novel therapeutic opportunities. STING agonists have shown considerable promise in immunotherapy for tumors and chronic viral infections, whereas STING inhibitors have paved the way for novel treatments of inflammatory diseases, including NAFLD, ALD, IBD, and liver fibrosis. The field faces both challenges and opportunities moving forward. Key areas for advancement include the development of cell-specific targeted delivery systems, identification of optimal therapeutic windows, exploration of refined regulatory mechanisms within the pathway, and the implementation of personalized treatments based on biomarkers. Effectively harnessing this dual-function pathway holds the potential for groundbreaking advancements in the treatment of various challenging liver and gastrointestinal diseases.
